# Ambulatory hernia repair: a study of 1294 patients in a single institution

**DOI:** 10.11604/pamj.2022.42.235.32863

**Published:** 2022-07-27

**Authors:** Mohamed Hajri, Dhafer Haddad, Haithem Zaafouri, Mouna Cherif, Alia Zouaghi, Nizar Khedhiri, Ali Mrabet, Anis Ben Maamer

**Affiliations:** 1 Department of General Surgery, Habib Thameur University Hospital, Tunis, Tunisia,; 2 Department of Public Health and Epidemiology, Faculty of Medicine of Tunis, University of Tunis El Manar, Tunis, Tunisia

**Keywords:** Surgery, ambulatory, hernia

## Abstract

**Introduction:**

ambulatory surgery is continuously expanding in global reach because of its several advantages. This study aimed to describe the experience of our department in outpatient hernia surgery, evaluate its feasibility and safety, and determine the predictive factors for failure of this surgery.

**Methods:**

we conducted a monocentric retrospective cohort study on patients who had ambulatory groin hernia repair (GHR) and ventral hernia repair (VHR) in the general surgery department of the Habib Thameur Hospital in Tunis between January 1^st^, 2008 and December 31^st^, 2016. Clinicodemographic characteristics and outcomes were compared between the successful discharge and discharge failure groups. A p-value of ≤ 0.05 was considered significant.

**Results:**

we collected data from the record of 1294 patients. One thousand and twenty patients had groin hernia repair (GHR). The failure rate of ambulatory management of GHR was 3.7%: 31 patients (3.0%) had unplanned admission (UA) and 7 patients (0.7%) had unplanned rehospitalization (UR). The morbidity rate was 2.4% while the mortality rate was 0%. On multivariate analysis, we did not identify any independent predictor of discharge failure in the GHR group. Two hundred and seventy-four patients underwent ventral hernia repair (VHR). The failure rate of ambulatory management of VHR was 5.5%: 11 patients (4.0%) had UA and 4 patients (1.5%) had UR. The morbidity rate was 3.6% and the mortality rate was zero. On multivariate analysis, we did not identify any variable predicting discharge failure.

**Conclusion:**

our study data suggest that ambulatory hernia surgery is feasible and safe in well-selected patients. The development of this practice would allow for better management of eligible patients and would offer many economic and organizational advantages to healthcare structures.

## Introduction

Ambulatory surgery is defined as surgery without an overnight hospital stay. In this type of surgery, patients are discharged on the day of the surgical operation. Apart from its advantages in terms of patient comfort and reduced risk of nosocomial infections, it is of great economic interest as it reduces the expenses related to conventional hospitalization [1]. The rate of ambulatory surgery in the world is increasing. In Tunisia, our experience began in 2008 at the Habib Thameur University Hospital in Tunis. The main surgical indications for ambulatory surgery in our centre are groin hernia repair (GHR), ventral hernia repair (VHR), laparoscopic cholecystectomy and proctology. This study aims to describe the experience of our department in outpatient hernia surgery, evaluate its feasibility and safety, and determine the predictive factors for failure of this surgery.

## Methods

**Study design:** we conducted a monocentric retrospective cohort study on patients who had ambulatory groin hernia repair (GHR) and ventral hernia repair (VHR) in the general surgery department of the Habib Thameur Hospital in Tunis between January 1^st^, 2008 and December 31^st^, 2016.

**Study setting and participants:** age ≥ 16 years, American Society of Anaesthesiologists (ASA) Grade I or II, the availability of an adult companion during the first 48 hours after surgery, a distance of 50 km or less from the hospital, and patient agreement and consent to the ambulatory procedure. The exclusion criteria were patients: on chronic anticoagulants, with allergies to anaesthetic drugs, with recurrent or strangulated hernias, with large ventral hernia (VH) with an expected parietal defect of >10 cm or those with the necessity of drainage.

**Study size:** we conducted this study in the first-day case surgery unit of our hospital. It is an isolated satellite structure located in our general surgery department. It includes a waiting room, an operating room, and two post-interventional monitoring rooms (PIMRs) with a total capacity of four patients, a restroom, a changing room, an office for the medical and paramedical staff and a sterilization room. All the patients included in this study were examined by a surgeon and evaluated by an anesthesiologist as part of the preanesthesia consultation. As part of counselling, the patient must be informed of the surgical procedure, the type of anesthesia and the peri-operative instructions. Patients were admitted into the hospital at 7 am. They were transferred to the operating room after being examined by the surgeon. The anesthesia protocol relied mainly on the anesthesiologist, who considered the ambulatory aspect of the procedure when choosing the appropriate anaesthetic drugs. After surgery, the patients were transferred to the PIMR. Before a patient could be safely discharged from the PIMR, a careful examination was conducted and the modified Aldrete score computed. This score assesses five parameters: respiration, blood pressure, consciousness, colour and activity level. Each parameter is scored 0, 1 or 2, and patients scoring nine or greater were eligible to be transferred to the restroom. The hospital discharge was carried out the same day as the procedure, before 7 pm. The necessary conditions for discharge were the stability of vital signs, a well-tolerated oral liquid diet, ambulation without dizziness, efficiency of the oral analgesia, a spontaneous micturition, the absence of nausea and vomiting, the patient's agreement for the discharge, the availability of an adult companion during the first 48 hours after surgery and discharge before 7 pm.

**Variables:** the analysis was based on the success or failure of the ambulatory procedure. The study compared two groups: a) The “successful discharge” group included patients who were operated on and discharged on the same day of surgery before 7 pm with no unplanned rehospitalization or postoperative re-operation, and b) The “discharge failure” group, which included: unplanned admissions (UAs) occurring before the patient's discharge from the hospital; unplanned rehospitalisations (URs) occurring after the patient's discharge during the 48 hours following surgery.

**Statistical methods:** statistical analysis was performed using SPSS 23.0 (Statistical Package for the Social Sciences software for Windows). We compared the two groups “successful discharge” and “discharge failure” with regard to their demographic characteristics, ASA classes, type of hernia, anaesthesia and surgical technique used, operative time, success or failure of discharge on the same day of surgery, and postoperative morbidity and mortality. Bivariate analysis was done with categorical data compared using the Chi-squared test, while continuous data were compared using the Student´s t-test. Significant variables in the literature were introduced into a multivariate logistic regression model to identify predictive factors for failure. A p-value of p ≤ 0.05 was considered significant.

## Results

**Participants**: a total of 1294 patients were included.

### Descriptive data

**Groin hernia repair:** one thousand and twenty patients underwent GHR. The mean age was 45.0 ± 10.2 years (17-88 years). The population was made up mainly of male patients (95%) with a male to female ratio at 19: 1. Eight hundred and twenty-seven patients (81.1%) were of ASA Grade I and 193 (18.9%) had an ASA Grade of II. Eight hundred and two patients (78.6%) had an inguinal hernia, 162 (15.9%) had an inguinoscrotal hernia and 56 (5.5%) had a femoral hernia. Seventy-two patients (7.1%) were operated under general anesthesia (GA), and 948 (92.9%) under spinal anesthesia. The Lichtenstein technique was performed in 518 patients (50.8%), the Bassini technique in 441 patients (43.2%), the Mac Vay technique in 44 patients (4.3%), the Rives technique in 12 patients (1.2%), and the Shouldice technique in five patients (0.5%). The Bassini technique was mainly used during the first years of the study, but since 2015 the Lichtenstein technique was exclusively used for inguinal hernias. The mean operation time was 51.0 ± 9.0 minutes (30-95 minutes). The success rate of the ambulatory procedure was 96.3%. Thirty-one patients (3.0%) had a UA, the causes for which were the following: postoperative pain (POP) in nine patients (29%), headache and vertigo in nine patients (29%), postoperative nausea and vomiting (PONV) in seven patients (22.6%), supporting problems in four patients (12.9%), and acute urinary retention in two patients (6.5%). Seven patients (0.7%) had a UR, the causes for which were the following: abdominal wall hematoma in three patients (42.8%), acute urinary retention in two patients (28.6%) and hematocele in two patients (28.6%). The failure rate of ambulatory management of GHR, the sum of UA and UR, was therefore 3.7%. On bivariate analysis, we found that the variables of age, GA, Lichtenstein technique and operation time were significantly more likely to lead to discharge failure in GHR patients (p < 0.001, p = 0.004, p < 0.001 and p < 0.001, respectively; Table 1). In multivariate analysis, we did not identify any variable predicting discharge failure in the GHR group. Complications occurred in 24 patients (2.4%). These consisted of abdominal wall hematoma in eight patients (33.3%), acute urinary retention in eight (33.3%), cutaneous abscess of the abdominal wall in five (20.8%), hematocele in two (8.4%) and hydrocele in one patient (4.2%). The death rate after GHR management was zero.

**Table 1:** comparison of “discharge failure” and “successful discharge” groups after groin hernia repair

Variables		Discharge failure	Successful discharge	P
Age (years)		56.0 ± 12.0	45.0 ± 10.0	<0.001
Gender	Male	35 (3.6%)	934 (96.4%)	0.432
	Females			
ASA Grade	ASA I	30 (3.6%)	797 (96.4%)	0.677
	ASA II	8 (4.1%)	185 (95.9%)	
Type of anaesthesia	GA	8 (11.1%)	64 (88.9%)	0.004
	Spinal anaesthesia			
Type of hernia	Inguinal hernia (IH)	27 (3.4%)	775 (96.6%)	0.068 (IH vs ISH)
	Inguinoscrotal hernia (ISH)	11 (6.8%)	151 (93.2%)	0.099 (ISH vs FH)
	Femoralhernia (FH)	0 (0.0%)	56 (100.0%)	0.317 (IH vs FH)
Surgical technique	Lichtenstein	33 (6.4%)	485 (93.6%)	<0.001(Lichtenstein vs Bassini)
	Bassini	5 (1.1%)	436 (98.9%)	
	Mac Vay	0 (0.0%)	44 (100.0%)	
	Rives	0 (0.0%)	12 (100.0%)	
	Shouldice	0 (0.0%)	5 (100.0%)	
Mean operative time (minutes)		57.0 ± 9.0	50.0 ± 8.0	<0.001

**Ventral hernia repair:** two hundred and seventy-four patients underwent VHR. The mean age was 43.7 ± 11.9 years (17-80 years). Ninety patients (32.8%) were male and 184 (67.2%) female, with a male to female ratio at 0.48: 1. Two hundred and ten patients (76.6%) were of ASA Grade I and 64 (23.4%) had an ASA Grade of II. One hundred and eighty patients (65.7%) had an umbilical hernia (UH) and 94 (34.3%) had an epigastric hernia (EH). All the patients were operated on under GA. One hundred and forty-eight patients (54.0%) underwent mesh repair, while 126 (46%) underwent suture repair. The mean operation time was 42.0 ± 8.0 minutes (20-65 minutes). The success rate of the ambulatory procedure was 94.5%. Eleven patients (4.0%) had a UA, the causes for which were the following: PONV in four patients (36.3%), POP in three (27.3%), headache and vertigo in two (18.2%) and supporting problems in two patients (18.2%). Four patients (1.5%) had a UR for the following reasons: abdominal wall hematoma in two patients (50.0%) and seroma in two patients (50.0%). The failure rate of ambulatory management of VHR was therefore 5.5%. On bivariate analysis, we found out that the variables of age, ASA Grade II and operation time were significantly more likely to lead to discharge failure in VHR patients (p < 0.001, p < 0.001 and p = 0.033, respectively; Table 2). On multivariate analysis, we did not identify any variable predicting discharge failure in the VHR group. Complications occurred in 10 patients (3.6%). These consisted of cutaneous abscess of the abdominal wall in four patients (60.0%), abdominal wall hematoma in two (20.0%) and seroma in two patients (20.0%). The death rate after VHR management was 0%.

**Table 2:** comparison of “discharge failure” and “successful discharge” groups after ventral hernia repair

Variables		Discharge failure	Successful discharge	P
Age (years)		61.0 ± 8.0	43.0 ± 11.0	p<0.001
Gender	Male	5 (5.5%)	85 (94.5%)	1.000
	Female			
ASA Grade	ASA I	4 (1.9%)	206 (98.1%)	p<0.001
	ASA II	11 (17.2%)	53 (82.8%)	
Type of hernia	Umbilical hernia (UH)	10 (5.5%)	170 (94.5%)	1.000
	Epigastric hernia (EH)	5 (5.3%)	89 (94.7%)	
Surgical technique	Meshrepair	8 (5.4%)	140 (94.6%)	1.000
	Suture repair	7 (5.6%)	119 (94.4%)	
Mean operative time (minutes)		46.0 ± 7.0	41.0 ± 8.0	0.033

## Discussion

These results suggest that ambulatory hernia surgery is feasible and safe. Nevertheless, appropriate patient selection is essential to achieve such results. The selection criteria are not just medical but are also related to environmental and psychosocial factors [2]. In the literature, inclusion criteria vary according to authors. Bringman set an age limit of fewer than 75 years [3]; other authors, such as Mamie and Metzger, set an age limit of under 65 years [4]; this should minimize failure rates, especially in elderly patients. There were no age limits for other series, such as by Kurzer [5], Sanjay [6] or Kark [7]. In our study, advanced age was not acontra indication; we included all patients whose age was over 16 years. Some series, such as by Arroyo [8] included patients with ASA Grade III. In the French Society of Anesthesia and Intensive Care (SFAR) recommendations, outpatient anaesthesia is preferentially intended for ASA Grade I and II patients. But some ASA Grade III patients can be eligible with some conditions: stabilized pathology under adapted treatment, negligible interferences between the intervention and the underlying pathologies and prior agreement between the surgeon and anesthetist [9]. In most studies, bilateral or recurrent hernias were not a limitation to outpatient management. According to some authors, such as Drissi and Kark, these two factors were not associated with a higher risk of failure of the ambulatory procedure [7,10-12]. In our study, patients under 16 years of age, patients with an ASA Grade ≥ III, on long-term anticoagulants or under a medical treatment requiring its interruption before surgery, patients who were allergic to the anaesthetic drugs, had large VH of ≥10 cm or recurrent and strangulated hernias were not included. In some series such as by Drissi or Hanes [13,14], the principal exclusion criteria were: strangulated hernias undergoing emergency surgery, large VH, large inguinoscrotal hernias and the necessity of drainage. According to SFAR, the specific exclusion criteria for ambulatory hernia procedures are: complicated hernias undergoing emergency surgery, bulky and/or incarcerated inguinoscrotal hernias and large VH [9]. Hernias requiring drainage and bulky VHs with a wall defect of >10 cm were excluded in our series.

The pre-anesthesia evaluation should be carried out several days before surgery. All anesthesia techniques can be performed, and there are no specific techniques for outpatient procedures, but drugs with a shorter duration of action are recommended [15]. Patients must undergo the same pre-operative monitoring and surveillance as those in inpatient care [16]. The surgical principles and techniques are the same as those performed in traditional inpatient surgery [1]. According to several authors, the procedure should be performed by an experienced surgeon or under the supervision of a senior physician [17]. In our study, all patients were operated on by a senior or under the supervision of a senior, aiming to reduce the operation duration and the morbidity rate in order to minimize the failure rate of outpatient management. The main surgical procedures performed in our day surgery unit are hernia surgery, proctology, laparoscopic cholecystectomy and lipoma surgery. We have only included in our work the hernia surgery that we performed exclusively by laparotomy. The surgical techniques varied according to the preferences of the surgeon. After surgery, patients are transferred to a PIMR, where careful and continuous monitoring is carried out [18]. The monitoring time varies between patients and depends mainly on the type and duration of anesthesia and the occurrence of intraoperative events [16]. The medical and surgical staff must carry out a standardized protocol for anticipatory control of POP and PONV [19-21]. Locoregional analgesia (LRA) is recommended. The surgeon should infiltrate the surgical wound with long-acting local anesthetics as soon as possible, as this will significantly reduce the incidence of POP [22,23]. Morphine use should be avoided as it can cause PONV and urinary retention [24]; it should be reserved for severe and resistant pain [15]. The use of NSAIDs significantly reduces PONV, particularly through opioid sparing [22,24]. A therapeutic approach combining intraoperative droperidol and dexamethasone, while reserving strong antiemetics such as setrons for curative treatment, is quite satisfying in this context [22,25].

Discharge from the PIMR is conditioned by several clinical monitoring parameters combined into scores, the most commonly used being the modified Aldrete score [26]. Once the patient has a score of 9 or higher, he/she can be transferred to the restroom [15,16]. In our study, we also used the modified Aldrete score. The surgeon and the anesthetist must approve the patient´s discharge after a medical evaluation, and a prescription can be delivered by one of the physicians of the structure [1]. The majority of authors agree that before discharge patients must be perfectly conscious, tolerate oral feeding, have spontaneous urination, do not require parenteral analgesia, and be able to walk on their own without assistance, with the mandatory presence of an adult companion [15,20,27-29]. In this context, the use of a predetermined score is recommended to facilitate this step. The most commonly used score is the PADSS “discharge” score, which considers vital signs (heart rate and blood pressure), ambulation, POP and PONV, the eventual occurrence of bleeding, and nutrition. A score of ≥ 9 allows the patient to be discharged [29]. By analyzing the different outpatient hernia surgery series, we found very encouraging results, with success rates ranging from 74 to 100% [10,12,17,30,31]. Drissi's multicentric study reported 6974 patients that underwent ambulatory GHR between 2011 and 2015, achieving a success rate of 96.4% [10]. A French study performed by Ngo on 242 patients undergoing ambulatory hernia surgery between June 2008 and October 2009 reported a success rate of 96.7% [30]. The failure rate of the ambulatory procedure is estimated by the rate of UAs and URs [10,17]. In our study, the failure rate was 3.7% in GHR patients and 5.5% in VHR patients, while the success rate was 96.3% in GHR patients and 94.5% in VHR patients.

The rate of UA ranges from 0% and 19% in the literature [7,10,17,30,32-34]. Ngo *et al*. reported a UA rate of 2.6% [30]. Drissi *et al*. included 6974 patients in their study and reported a UA rate of 3.6%. The main causes of failure were headache and vertigo (20.6%), POP (15.7%), acute urinary retention (9.9%), supporting problems (7.6%), bleeding (4.6%), PONV (4.2%), and late discharge from the operating room (3.4%) [10]. A Belgian study published in 2018 by Van Caelenberg *et al*. [34], including 5156 patients that underwent ambulatory surgery, had a missed discharge rate of 2.89%. The main causes for UA were: socioeconomic and supporting problems (distance from the hospital, companion problems, late discharge from the operating room, etc.) in 45.52%, POP in 11.72%, bleeding and abdominal wall hematoma in 10.34%, acute urinary retention in 3.3%, PONV in 2% and medical causes in 5.52% (pulmonary embolism, pneumonia, epilepsy, syncope, etc). The UR rate ranges from 0% to 9% in the literature [17,30,33-37]. In Ngo's series, a rate of 0.7% of rehospitalizations was observed [30]. A multicentre Finnish study by Mattila *et al*. including more than 6500 patients having outpatient surgery in 2009 for several surgical pathologies including hernia, found a rate of post-discharge consultations of 5.9% and a UR rate of 0.7% [35].The different UR causes in the literature include: bleeding and abdominal wall hematoma, acute bowel obstruction, POP, acute urinary retention, headache and vertigo, fever and thromboembolic events [5,6,8,35,38-42].

To improve results and decrease the rates of UA and UR, several series have studied the predictive factors of failure of the ambulatory procedure [10,33,34,43-45]. Drissi *et al*. [10] analyzed these factors through a multivariate statistical study and concluded that an ASA Grade ≥ III, bilateral and/or strangulated hernias and spinal anesthesia were predictive factors for failure. Van Caelenberg *et al*. [34] found a failed discharge rate of 2.89%, related to longer operative duration, late discharge from the operating room and ASA Grade ≥ III. In Whippey's Canadian study published in 2013 [33], including 20,657 patients undergoing ambulatory surgery, the failed discharge rate was 2.67%. Predictive factors for failed discharge were: duration of surgery of more than one hour, ASA Grade ≥ III, advanced age (>80 years), and a body mass index (BMI) between 30 and 35 kg/m^2^. On the other hand, active smoking was associated with a reduced probability of failed discharge. Our study did not observe any statistically significant variable as a predictor of discharge failure by multivariate analysis. Our data are in agreement with those in the literature. We summarize in Table 3 the results of the different series according to morbidity and success or failure of the ambulatory procedure. The benefits of outpatient hernia surgery are currently recognized, as shown by the very high satisfaction rates of patients operated using this mode, which are over 90% in most series [1,23,29,46-48]. Ambulatory management decreases the rate of nosocomial infections. Indeed, early patient discharge reduces the probability of being exposed to infection [1]. It also allows for more comfortable care, leading to fewer daily changes in the patient's life and environment in comparison to conventional surgery [49]. Ambulatory surgery is also of major economic benefit. This is partly due to reduced hospitalization expenses, such as parenteral drug prescriptions and hygiene and maintenance costs [1,50]. It also allows for better hospital resource management by devoting full hospital bed capacity and nursing staff energy to patients with more serious pathologies [50].

**Table 3:** results of the different series according to morbidity and success or failure of ambulatory procedure

Series	N	Morbidity	Mortality	Success rate	Unplanned admissions (UAs)	Unplanned rehospitalisations (URs)
**Ngo *et al***.	242	3.3%	0.0%	96.7%	2.6%	0.7%
**Drissi *et al***	6974	7.3%	-	96.4%	3.6%	-
**Van Caelenberg *et al***.	5156	-	-	-	2.9%	-
**Mattila *et al***.	6659	-	-	-	5.9%	0.7%
**Whippey *et al***.	20657	-	-	-	2.7%	-
**Majholm *et al***.	57709	-	0.0004%	-	-	1.2%
**De Lange *et al***.	3284	5.9%	0.12%	-	-	-
**Our serie (groin hernia repair)**	1020	2.4%	0.0%	96.3%	3.0%	0.7%
**Our serie (ventral hernia repair)**	274	3.6%	0.0%	94.5%	4.0%	1.5%

## Conclusion

The ambulatory practice has become the reference procedure in hernia surgery when the necessary safety conditions are provided. The development of advanced surgical techniques and the advent of short-acting anesthetic drugs have contributed to the expansion of this practice. In terms of quality of care, the excellent patient satisfaction rate confirms the acceptability of this surgery. Our study data suggest that ambulatory hernia surgery is feasible and safe in well-selected patients. The development of this practice would allow for better management of eligible patients and would offer many economic and organizational advantages to healthcare structures.

### What is known about this topic

- Ambulatory surgery has several advantages in terms of patient comfort, reduced risk of nosocomial infections and greater cost benefit;
- The rate of ambulatory surgery in the world is increasing;
- The development of advanced surgical techniques and the advent of short-acting anesthetic drugs have contributed to the expansion of this practice.

### What this study adds

- Ambulatory hernia surgery is feasible and safe in well-selected patients;
- A re-evaluation of the inclusion criteria could allow a significant number of patients to benefit from this surgery;
- We have described our experience by reporting one of the largest series of ambulatory hernia surgery in Africa, and encourage developing countries to promote this type of surgery considering its economic benefit.
